# May the Force Not Be With You During Culture: Eliminating Mechano-Associated Feedback During Culture Preserves Cultured Atrial and Pacemaker Cell Functions

**DOI:** 10.3389/fphys.2020.00163

**Published:** 2020-03-20

**Authors:** Noa Kirschner Peretz, Sofia Segal, Yael Yaniv

**Affiliations:** Biomedical Engineering Faculty, Technion Israel Institute of Technology, Haifa, Israel

**Keywords:** 2, 3-butanedione-monoxime, blebbistatin, mechanics, sinoatrial node, ventricular

## Abstract

Cultured cardiomyocytes have been shown to possess significant potential as a model for characterization of mechano-Ca^2+^, mechano-electric, and mechano-metabolic feedbacks in the heart. However, the majority of cultured cardiomyocytes exhibit impaired electrical, mechanical, biochemical, and metabolic functions. More specifically, the cells do not beat spontaneously (pacemaker cells) or beat at a rate far lower than their physiological counterparts and self-oscillate (atrial and ventricular cells) in culture. Thus, efforts are being invested in ensuring that cultured cardiomyocytes maintain the shape and function of freshly isolated cells. Elimination of contraction during culture has been shown to preserve the mechano-Ca^2+^, mechano-electric, and mechano-metabolic feedback loops of cultured cells. This review focuses on pacemaker cells, which reside in the sinoatrial node (SAN) and generate regular heartbeat through the initiation of the heart’s electrical, metabolic, and biochemical activities. In parallel, it places emphasis on atrial cells, which are responsible for bridging the electrical conductance from the SAN to the ventricle. The review provides a summary of the main mechanisms responsible for mechano-electrical, Ca^2+^, and metabolic feedback in pacemaker and atrial cells and of culture methods existing for both cell types. The work concludes with an explanation of how the elimination of mechano-electrical, mechano-Ca^2+^, and mechano-metabolic feedbacks during culture results in sustained cultured cell function.

## Introduction

Cardiac physiology research aims to understand how the heart works under both healthy and pathophysiological conditions, since the prevention and treatment of cardiovascular abnormalities, including arrhythmias, is the most critical goal of biomedical researchers. A top-to-bottom approach, i.e., parallel performance of *in vivo* experiments and *in vitro* cardiac tissue and single-cell experiments, is often taken to achieve these goals. Animals are the most favorable model, as the production of human-like cells and tissues remains a challenge. In order to understand true cardiac physiology, the mammalian cells and tissues must be maintained under conditions similar to those prevailing in the body, with temperature and extracellular conditions being crucial for physiological cell and tissue functioning. More specifically, to explore the mechano-electrical, mechano-Ca^2+^, and mechano-metabolic feedbacks that determine heart function (e.g., Braunwald, 1971; Bers, 2008; Quinn, 2014; Kamoun et al., 2018), one of the most fascinating branches of cardiac physiology, the ability of spontaneously beating cells to self-contract at their physiological frequency or of electrically triggered cells to remain quiescent in the absence of electrical stimulation and to respond to posed physiological frequency stimuli, must be preserved.

Recently, new genetic manipulation techniques, including Förster resonance energy transfer (FRET), have been applied to measure contractile filament activity, Ca^2+^ cycling, and metabolite dynamics (Warrier et al., 2005; Lu et al., 2013; Kioka et al., 2014; Li et al., 2016; Musheshe et al., 2018). Most of these experiments can only be performed in culture. Thus, a sustainable, viable and physiological-like culture method is crucial. Cardiac cell culture protocols ensuring maintenance of cell morphology, cell quiescence in culture, response to electrical stimulation at a physiological rate (or spontaneously beating under physiological conditions), and preservation of Ca^2+^ cycling and bioenergetic function are viewed as successful protocols. However, currently, the majority of cultured cardiomyocytes exhibit impaired electrical, mechanical, biochemical, and metabolic functions.

**FIGURE 1:**
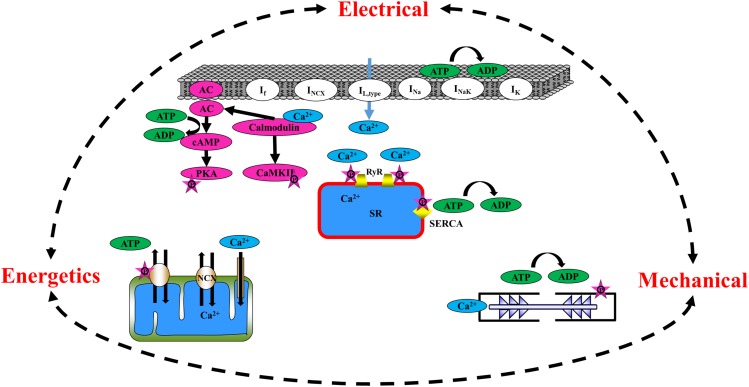
Schematic illustration of the major players in mechano-electrical, mechano-Ca^2+^, and mechano-metabolic feedbacks.

This review focuses on pacemaker cells, which reside in the sinoatrial node (SAN) and generate the heartbeat by initiating the heart’s electrical, metabolic, and biochemical activities. In parallel, it places emphasis on atrial cells, which are responsible for bridging the electrical conductance from the SAN to the ventricle. More specifically, the review provides a summary of the main mechanisms responsible for mechano-electrical, Ca^2+^, and metabolic feedback in pacemaker and atrial cells (Figure 1) and of the existing culture methods for each cell type. The work concludes with an explanation of how the elimination of mechano-electrical, mechano-Ca^2+^, and mechano-metabolic feedbacks during culture leads to sustained cultured cell function.

## Electro-Ca^2+^-Metabolic-Mechanical Feedback in Pacemaker and Atrial Cells

The heart is assembled from different cell types that, together, determine its function. The atria are comprised of two of the four chambers of the heart and are situated above the two ventricles. The right atrium contains the SAN, the heart’s primary pacemaker, which initiates heart electrical activity (Monfredi et al., 2010) by generating an action potential (AP) that spreads across both atria, inducing their contraction and forcing the blood they hold into their corresponding ventricles. The pacemaker cell beating rate is controlled by internal pacemaker cell clocks and through brain signaling that activates receptors on the pacemaker cell membrane (Vinogradova et al., 2002). During early depolarization, prior to the burst of an AP in the pacemaker cell, Ca^2+^ is spontaneously released from the sarcoplasmic reticulum (SR) through the ryanodine receptor channels (RyR2), close to the cell membrane, subsequently activating the Na^+^/Ca^2+^ exchanger (NCX) (Bogdanov et al., 2001). The inward current through the NCX, together with the funny current channels (I_f_), which are highly expressed in pacemaker cells (DiFrancesco and Ojeda, 1980), prompts the surface M-clock, an ensemble of sarcolemmal electrogenic molecules, to generate an AP (Lyashkov et al., 2018). The AP stimulates the opening of Ca^2+^ channels on the cell membrane (L-type Ca^2+^ channels, I_Ca,L_), hence enabling Ca^2+^ ion release into the cells, which arouses the release of Ca^2+^ ions from the SR into the cytosol. The SR, the organelle that initiates this chain of events, is known as the Ca^2+^ clock. Together, the M and Ca^2+^ clocks create a coupled-clock system (Lakatta et al., 2010). The crosstalk between the two clocks is dictated by Ca^2+^ and by secondary Ca^2+^-activated mechanisms [reviewed in Yaniv et al. (2015b)]. More specifically, Ca^2+^ activates calmodulin, which, in turn, regulates the adenylyl cyclase (AC) and Ca^2+^/calmodulin-dependent protein kinase II (CaMKII) function. AC catalyzes the conversion of adenosine triphosphate (ATP) to cyclic adenosine monophosphate (cAMP), which exposes the phosphorylation sites on the protein kinase A (PKA) molecules. PKA signaling cascades, together with CaMKII, act on both SR [phospholamban (PLB) and the RyR2] and M-clock proteins (L-type Ca^2+^ channels and K^+^ channels). In parallel, AC-cAMP signaling regulates the binding of cAMP molecules to the funny channel. Taken together, pacemaker electro-Ca^2+^ feedback is controlled by intrinsic signaling mechanisms originating at multiple cellular (surface membrane), intracellular (phosphorylation cascades, SR, the mitochondria), and heart-brain (neurotransmitter or hormonal stimulation of surface membrane receptors) locations. cAMP/PKA signaling also links the pacemaker electro-Ca^2+^ feedback to energetics. To maintain proper electrical activity in pacemaker cells, ATP is supplied to the cell in order to produce cAMP, maintain ionic homeostasis, and pump Ca^2+^ into the SR. It has been shown that Ca^2+^-dependent activation of cAMP/PKA signaling within pacemaker cells links ATP utilization and its production in the mitochondria; when the spontaneous AP firing rate is decreased by drugs that lead directly or indirectly to cAMP/PKA reduction, ATP in the pacemaker cells is depleted (Yaniv et al., 2011). Similar results were found when CaMKII signaling was eliminated using pharmacological drugs (Yaniv et al., 2013a). When the ATP demand increases above basal levels, the mitochondrial cAMP/PKA-CaMKII signaling and mitochondrial Ca^2+^ work together to increase the ATP supply to match the increase in energetic demand (Yaniv et al., 2013b). An additional electro-energetics link in pacemaker cells has been shown by changes in SR Ca^2+^ loading and spontaneous Ca^2+^ release in response to changes in mitochondrial Ca^2+^ flux, all of which translate into changes in cell automaticity (Yaniv et al., 2012). Similar evidence was found upon genetic manipulation of the Ca^2+^ influx channel (Wu et al., 2015). Taken together, the role of pacemaker cell mitochondria is not limited to ATP production alone, but rather, extends to the cells’ coupled-clock system and its influence on the AP firing rate (i.e., reverse electro-energetic feedback). Finally, electrical-mechanical feedback also regulates pacemaker cells. Although questioned in the past, it has been firmly established that pacemaker cells possess sarcomeres, the basic contractile unit (Yaniv et al., 2011). Evidence for such feedback was demonstrated in heart failure patients presenting a distended atrial wall (the location of the SAN tissue) as a result of increased venous return post-exercise and saline loading (Andersen et al., 2015). Another study showed a strong relationship between elevated right atrial pressure and pacemaker cell dysfunction (Paul et al., 1998). Extensive research also showed that mechano-electrical feedback exists in pacemaker cells and SAN tissue. An increase in the beating rate in response to mechanical stretch of the SAN tissue (Lange et al., 1966; Kamiyama et al., 1984) and of pacemaker cells (Kohl et al., 1999) has been documented, demonstrating that the heart’s positive stretch-induced chronotropic response is caused by mechanisms within the SAN tissue. However, the main regulators of this feedback have not been fully elucidated.

Propagation of the AP from the SAN ignites AP in the atria and leads to the opening of L-type Ca^2+^ channels, which induce the release of Ca^2+^ ions from the SR. The majority of the Ca^2+^ released attaches to the sarcomeres, with only a small portion entering the mitochondria (Bootman et al., 2006; Maack and O’Rourke, 2007). Similarly to pacemaker cells, atrial cells express AC type 1 and 8 (Collins and Terrar, 2012). Intracellular Ca^2+^ binds to calmodulin, which activates AC-cAMP/PKA molecules, which, in turn, increases phosphorylation activity in the cell. The cAMP produced by Ca^2+^-stimulated ACs regulates the L-type Ca^2+^ channel and, subsequently, the calcium-induced calcium release (CICR) in the atria (Collins and Terrar, 2012). PKA also serves as a regulator of electro-Ca^2+^ feedback in atrial cells via its effect on cell membrane channels [L-type Ca^2+^ channels (Trautwein and Hescheler, 1990), K^+^ channels (Freeman et al., 1992), Ca^2+^ storage within the SR (Toyofuku et al., 1993), and RyR2 (Takasago et al., 1989)]. T-tubules are extensions of the cell membrane that penetrate into the center of cardiac cells. They permit rapid transmission of APs into the cell and play an important role in regulating cellular Ca^2+^ concentrations. By synchronizing Ca^2+^ release throughout the cell (Hong and Shaw, 2017) and shifting the electro-mechanical feedback to occur in the peripheral areas of the cell, t-tubules allow cardiac cells to contract more forcefully. Atrial cells have a different t-tubule system than ventricular cells; they are termed transverse-axial tubules (TATs) and present as heterogenous sarcolemmal membrane invaginations that appear near the z-lines and assist in atrial cell contraction (Kirk et al., 2003; Hund and Mohlerp, 2016; Yue et al., 2017). When an AP is initiated, the atrial cells activate intracellular Ca^2+^ release and sarcomeric contraction through the TAT junctions (Brandenburg et al., 2018). The existence of the TATs contributes to near-synchronous Ca^2+^ transients and to synchronous subcellular depolarization and intracellular Ca^2+^ release. TATs may lead to region-specific electro-mechanical coupling and heterogeneous atrial contraction (Hund and Mohlerp, 2016; Yue et al., 2017; Brandenburg et al., 2018). There is evidence of dense and well-developed TATs in both small and large animals (Kirk et al., 2003; Glukhov et al., 2015; Yue et al., 2017; Brandenburg et al., 2018). In addition, studies have shown the existence of an atrial t-tubule system in larger mammals, such as sheep and humans (Richards et al., 2011; Trafford et al., 2013). As in the ventricles, which have a vast t-tubular system, Ca^2+^ release in atrial cells occurs simultaneously in both the central and peripheral areas of the cells. A highly efficient energy supply-and-demand matching process is essential to coordinate atrial mechano-metabolic feedback cues during permanent changes in cardiac workload. In response to an increase in electrical stimulation, frequency, or Ca^2+^ transient amplitude, Ca^2+^ accumulates in the atrial mitochondria and then activates enzymes participating in ATP production (Harada et al., 2017). It is not known whether cAMP/PKA and CaMKII signaling regulate ATP production in atrial cells, as has been seen in pacemaker cells.

In summary, cAMP and PKA signaling, together with SR and mitochondrial Ca^2+^, are important regulators of mechano-electrical, mechano-Ca^2+^, and mechano-metabolic feedback loops (Figure 1). Thus, physiologically relevant measurements of these signals are necessary to gain a comprehensive understanding of their underlying mechanisms and role. As described above, such measurements are usually conducted on cultured atrial or pacemaker cells that are placed in a sustainable culture medium for a period of at least 24–48 h.

## Previous Culture Methods of Pacemaker Cells

Only a small number of labs have successfully established physiologically relevant methods for pacemaker cell culture. The first reported method utilized rabbit pacemaker cells (Liu et al., 1996), and while it shed light on the ionic current characteristics, it compromised several ionic channels and decreased the spontaneous beating rate. A later study from the same group demonstrated stable electrical activity in cultured rabbit pacemaker cells, but also showed a reduction in ionic currents (Muramatsu et al., 1996) in comparison to fresh cells. By modifying the culture method, Yang et al. (2012) caused pacemaker cells to generate spontaneous AP firing and rhythmical beating. However, the beating rate and the PKA-dependent phosphorylation level of PLB at site Ser^16^ and RyR2 at site Ser^2809^ were reduced in comparison to fresh cells. In addition, decreased levels of type 2 regulator of G-protein signaling (RGS2) and consequently dampened AC-cAMP/PKA signaling, were observed in comparison to fresh cells.

## Previous Culture Methods of Atrial Cells

Several studies have previously been conducted to characterize intrinsic atrial mechanisms (excluding heart-brain signaling) under culture conditions. In the first study, rabbit atrial cells remained viable in culture for up to 5 days (Gilliam et al., 1989). Yet cell morphology was not maintained; cells transformed from a rod-like to a spherical shape. Two additional studies (Wang et al., 1999; Rinne et al., 2010) also preserved rabbit atrial cell viability in culture for up to 48 h and showed cell stability following adenoviral infection. However, neither group was able to stimulate the cells at a rate above 0.5 Hz, a rate very far from the natural rabbit pacemaker cell beating rate of ∼3 Hz. In two additional studies on rabbit atrial cells (Hohendanner et al., 2015a, b), the researchers achieved similar results. Culture of atrial cells derived from other mammals has also been achieved. In one of the studies, rat atrial cells maintained a spindle-like morphology for a period of 3–7 days (Doupnik et al., 2001). In addition, expression of the acetylcholine-elicited inwardly rectifying potassium current (I_K,Ach_) proteins was consistent with data collected from similar cells. However, functional data of the cultured cells were not shown. Finally, human atrial cells in culture (Feng et al., 1996) survived for up to 5 days, but, within 24 h, the cells assumed an oval-like shape. The voltage dependence, kinetics, and density of the sodium current were maintained in culture compared to control. While the activation properties (kinetics and voltage dependence) of the potassium transient outward current were not altered, steady potassium outward current density (current normalized to cell capacitance) was reduced in cultured compared to fresh cells. All changes in ionic currents occurred within 24 h of culture and were not altered for 4 days.

## Eliminating Mechano-Associated Feedback During Culture Preserves Ventricular Cell Function in Culture

Soeno et al. (1999) suggested that because the myosin-actin interaction is important for the initial phase of myofibrillogenesis, eliminating this interaction may improve the quality of the culture methods. Indeed, upon inhibition of this interaction in primary skeletal muscle cultures by application of 2,3-butanedione-monoxime (BDM), an inhibitor of myosin ATPase, culture profiles improved, as manifested by maintained cell viability and morphology. Similarly, Kivistö et al. (1995) found that BDM-treated rat ventricular myocytes maintained their shape and AP parameters and showed increased survival rates. Later, Hall et al. (Hall and Hausenloy, 2016) showed that addition of BDM to cultured mouse ventricular myocytes also reduced the number of spontaneous contractions per minute, increased survival rates, and reduced oxygen consumption. When supplementing mouse ventricular cell cultures with BDM, Ackers-Johnson et al. (2016) noted that cells survived in culture for a period of up to 7 days and retained their morphology, functionality, and Ca^2+^ handling, with optimal results observed after 2–3 days of culture. BDM has been shown to reduce the attachment of ATP to myosin, thereby reducing energy consumption in the cell (Ostap, 2002) and preserving the cellular phosphorylation status (Segal et al., 2019). Taken together, elimination of mechano-associated feedback mechanisms, specifically energetics and other phosphorylation-dependent loops, preserves cell function in culture.

Studies have indicated that BDM is a largely non-specific agent (Ostap, 2002), with effects on L-type Ca^2+^ channel conductance (Gwathmey et al., 1991) and on I_K,s_ maximal conductance (Coulombe et al., 1990), and is associated with increased phosphatase activity (K_pp__1_) (Stapleton et al., 1998), and Ca^2+^ releases through RyR2 (Gwathmey et al., 1991). Due to the side effects of BDM, Kabaeva et al. (2008) sought to test other substances that eliminate mechano-associated feedbacks. Upon application of blebbistatin, to eliminate myofilament contraction in mouse ventricular myocytes during culture, the viability, membrane integrity, and morphology of ventricular myocytes were significantly increased as compared to cultures treated with BDM. Most importantly, the use of blebbistatin improved the efficiency of adenovirus-mediated gene transfer in the cultured myocytes compared to BDM (Kabaeva et al., 2008). Based on this study, use of blebbistatin to eliminate mechano-feedback has significant beneficial effects on the long-term usability of adult myocytes in primary culture, as it reduces ATP production (Kovács et al., 2004; Watanabe et al., 2010) and maintains a stable phosphorylation state in the cultured cardiomyocytes (Reddy et al., 2018; Segal et al., 2019) while avoiding the undesirable effects of BDM on adult myocytes.

## Eliminating Mechano-Associated Feedbacks Preserves Cultured Pacemaker Cell Function

**FIGURE 2:**
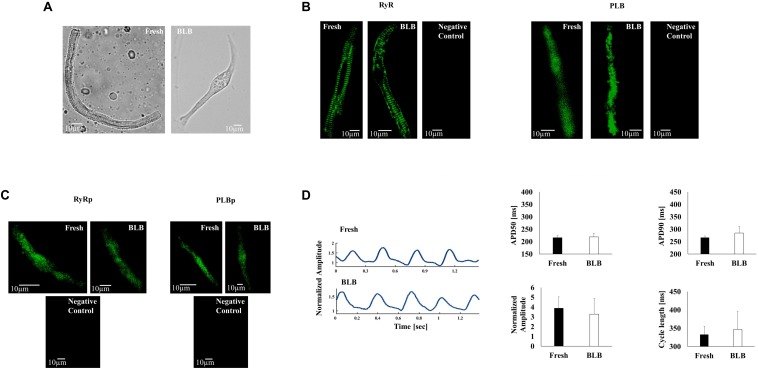
Pacemaker cell culture. **(A)** Representative examples of fresh and cultured pacemaker cells. **(B)** Representative examples of ryanodine receptor and PLB immunolabeling in fresh and cultured pacemaker cells (No first antibody was added to the negative control). **(C)** Representative examples of the phosphorylation status immunolabeling of the ryanodine receptors and SERCA in fresh and cultured pacemaker cells. **(D)** Representative examples of action potential in fresh and cultured pacemaker cells and average data for action potential characteristics (APD50, APD90, normalized amplitude, and cycle length). Data are from Segal et al. (2019). Animals were treated in accordance with the Technion Ethics Committee. The experimental protocols were approved by the Animal Care and Use Committee of Technion (Ethics number: IL-118-10-13).

The first attempt to eliminate mechano-feedback during culture of mouse pacemaker cells (St. Clair et al., 2015) used BDM, which yielded viable and spontaneously beating pacemaker cells within 48 h. In addition, cell morphology, AP firing rate, and two tested ionic current properties were preserved. Moreover, the group successfully genetically manipulated the cells in culture, thereby expanding the range of experimental techniques available for studying the physiological and functional properties of pacemaker cells. However, these experiments were performed on mouse pacemaker cells, which are inferior to rabbit pacemaker cells, which exhibit Ca^2+^ cycling similar to that of human cells. Further, Ca^2+^ cycling and phosphorylation activity were not measured, and thus, it remains unknown whether they are maintained in culture and whether the electro-Ca^2+^ feedback is maintained. Finally, no mechanistic explanation was provided regarding the observed effects of BDM on pacemaker cells. A recent study was designed to address these open questions (Segal et al., 2019) and to characterize the electro-Ca^2+^ feedback. The study compared the effect of blebbistatin and BDM on rabbit pacemaker cells in culture and sought to uncover the mechanisms underlying improved cultured pacemaker cell function upon elimination of mechano-associated feedback. BDM and blebbistatin treatment had identical effects on cell morphology (shape and area) (Figure 2A), and PLB and RyR2 densities and their phosphorylation levels, as well as on adenoviral transfection efficiency (Figures 2B,C). Blebbistatin-supplemented pacemaker cell cultures maintained their AP firing rate and global and local Ca^2+^ properties over 48 h (Figure 2D). In contrast, BDM suppressed these properties. Thus, to explore electro-Ca^2+^ feedback in cultured pacemaker cell, blebbistatin should be preferred over BDM. By using tetramethylrhodamine (TMRM) intensity per cell as an indicator of mitochondrial density, the group demonstrated that the addition of either mechanical feedback inhibitor preserved the mitochondrial density. Yet, as mitochondrial function was not assessed, it is not clear if this culture medium is appropriate for functional mechano-metabolic studies. Using a computational model, the researchers showed that reduced cellular energy consumption following the addition of a contraction inhibitor, together with the maintenance of phosphorylation activity, allowed for the preservation of cell characteristics and function under culture conditions. Moreover, the model showed that the decreased spontaneous AP firing rate was due to non-specific BDM side effects, which involved an indirect decrease in I_NCX_ and a direct decrease in the L-type Ca^2+^ currents. The model further confirmed that blebbistatin is superior to BDM in contraction elimination in culture and is a more optimal substance for preserving mechano-Ca^2+^ feedback. The question was raised of how BDM and blebbistatin preserved the electro-Ca^2+^ feedback in cultured pacemaker cell. The authors found that by eliminating contraction, phosphorylation activity is preserved and energy is reduced. Because PKA (Yaniv et al., 2015a; Behar et al., 2016) and CaMKII (Yaniv et al., 2013a) activities correlate with AP firing rates and affect electro-Ca^2+^ feedback, maintaining these signalings is essential for pacemaker function. In previous works, it was shown that Ca^2+^ and posttranslational modification signaling are the major mechanisms that maintain ATP supply to demand in pacemaker cells (Yaniv et al., 2011, 2013a,b). Thus, a reduction in these signaling cascades during culture may limit the ATP supply, consequently reducing the spontaneous beating rate.

A similar approach using BDM was applied for culturing human SAN and atrial tissue cardiac slices (Kang et al., 2016). The cardiac slice exhibited physiological automaticity similar to that of a normal human heart rate. Moreover, AP duration and Ca^2+^ transient regions were maintained in these cardiac slices. Importantly, these cardiac slices have the potential to be transfected with an adenovirus and express exogenous protein for a period of up 96 h.

## Elimination of Mechano-Associated Feedbacks During Culture Preserves Cultured Atrial Cell Function

**FIGURE 3:**
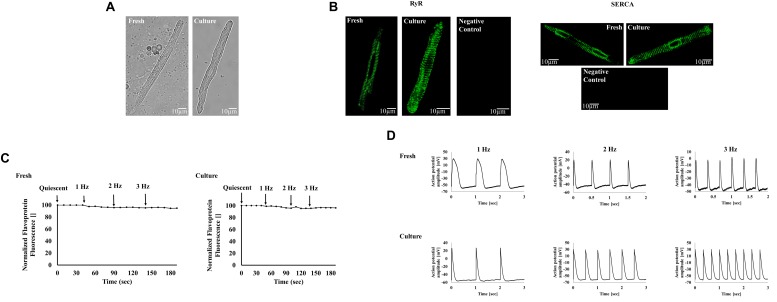
Atrial cell culture. **(A)** Representative examples of fresh and cultured atrial cells. **(B)** Ryanodine and SERCA immunolabeling for fresh and cultured atrial cells’ structures. **(C)** Representative examples of fresh and cultured atrial cell flavoprotein auto-fluorescence in response to increasing electrical stimulation (1–3 Hz). **(D)** Representative examples of fresh and cultured AP in atrial cells at 1–3 Hz of external electrical stimulation. Data from Kirschner-Peretz et al. (2017). Animals were treated in accordance with the Technion Ethics Committee. The experimental protocols were approved by the Animal Care and Use Committee of Technion (Ethics number: IL-118-10-13). **(C)** is reproduced from Kirschner-Peretz et al. (2017) with permission.

The first attempt to eliminate mechano-feedbacks in cultured atrial cells was documented in Kirschner-Peretz et al. (2017). Their work tested the effect of BDM on the biophysical, functional, and bioenergetic characteristics of cultured rabbit atrial cells. After 24 h in culture, their morphology and volume were similar to those of freshly isolated cells (Figure 3A). In the absence of BDM, the cultured atrial cells failed to maintain their morphology in comparison to fresh cells and could not be electrically paced at any stimulation rate. Importantly, the cells were successfully infected by an adenovirus by using exogenous proteins. Moreover, the mechano-Ca^2+^ feedback was sustained, with Ca^2+^ transients and local Ca^2+^ releases and intact RyR2 and SERCA structures similar to those of fresh cells (Figure 3B). In addition, assessment of the electro-metabolic feedback indicated that the energetic state of the cells remained constant in response to external electrical pacing at a physiological rate, a response similar to that of freshly isolated cells (Figure 3C). Finally, mitochondrial density, measured by TMRM average intensity, was unaffected by the inhibitor. Regarding the mechano-electrical feedback, the ability to undergo external electrical stimulation at frequencies of 1–3 Hz was maintained in the cultured cells; AP parameters were similar between fresh and cultured cells (Figure 3D), as was t-tubule density. Note that the authors tested the effect of BDM only. While electrical-Ca^2+^ and electrical-energetic feedbacks circuits were maintained over 24 h in culture, for longer culture periods, blebbistatin may prove superior to BDM. Taken together, the elimination of electro-mechanical feedback enables cultured atrial cells to maintain physiological electrical activity and, thus, electrical-Ca^2+^ and electrical-energetic feedback mechanisms.

### Summary

Optimized culture media are the key to the exploration of physiological electrical-Ca^2+^ and electrical-metabolic feedback mechanisms. Eliminating contraction during culture is critical for measuring mechano-electric, mechano-Ca^2+^, and mechano-metabolic feedbacks. Appropriate culture methods provide an important platform for further understanding energetic, biochemical, and electrical regulatory feedbacks in healthy pacemaker and atrial cells, which could potentially provide insights into their relations to the development and maintenance of cardiac diseases involving atrial and pacemaker dysfunction.

## Author Contributions

All authors listed have made a substantial, direct and intellectual contribution to the work, and approved it for publication.

## Conflict of Interest

The authors declare that the research was conducted in the absence of any commercial or financial relationships that could be construed as a potential conflict of interest.
